# Body Image, Emotional Eating and Psychological Distress among Bariatric Surgery Candidates in Israel and the United States

**DOI:** 10.3390/nu12020490

**Published:** 2020-02-14

**Authors:** Shulamit Geller, Sigal Levy, Ofra Hyman, Paul L. Jenkins, Subhi Abu-Abeid, Gil Goldzweig

**Affiliations:** 1 School of Behavioral Sciences, the Academic College of Tel Aviv-Yaffo, 14, Rabenu Yeruham St, Tel Aviv-Yaffo 6818543, Israel; giligold@mta.ac.il; 2 Statistics Education Unit, the Academic College of Tel Aviv, Tel Aviv-Yaffo 6818543, Israel; levy@mta.ac.il; 3 Outpatient Psychiatry, Bassett Medical Center, Cooperstown, NY 13326-1394 USA; ofrahyman@gmail.com; 4 Bassett Healthcare Research Institute, Bassett Medical Center, Cooperstown, NY 13326-1394, USA; paul.jenkins@bassett.org; 5 Bariatric Surgery Unit, General Surgery Division, the Tel Aviv Sourasky Medical Center, Tel Aviv-Yaffo 6423906, Israel; subhia@tlvmc.gov.il

**Keywords:** bariatric surgery, psychological distress, body image dissatisfaction, emotional eating, cross cultural differences

## Abstract

Background: The present study aimed to examine the relations between body image dissatisfaction (BID) and psychological distress variables among bariatric surgery candidates from two distinct cultures in Israel and in the United States. Methods: A sample of consecutive pre-surgical bariatric candidates was recruited from a Bariatric Center in Israel (*N* = 114) and a Bariatric Center in the Unites States (*N* = 81). Body image dissatisfaction (BID-BSQ8), suicidal ideation (SBQ-R), depressive symptoms (PHQ-9), anxious symptoms (PHQ-7), and emotional eating behaviors (EES), were measured. Mediation models were assessed using path analysis. Results: BID was positively correlated with suicidality, depression, and anxiety in both samples. The relations between BID depression and anxiety were mediated by emotional eating in both cultures. However, the relation between BID and suicidality that was mediated by emotional eating in the Israeli sample, was reflected in a direct link between BID and suicidality in the US sample. Conclusion: Our findings confirm the adverse effect of BID on psychological distress among surgery candidates in both cultures, emphasizing the intercultural similarities related to emotional eating behavior. Physicians and other health professionals are encouraged to be more attentive to this specific behavior.

## 1. Introduction

Obesity has become increasingly prevalent throughout the world [1]. Approximately one in every seven people is obese, totaling over half a billion people worldwide [2], and obesity has, thus, become a leading public health challenge. 

Bariatric surgery is a treatment proven to induce weight loss and to reduce the adverse effect of obesity-related physical comorbidity [3]. Patients who opt for bariatric surgery suffer a wider variety of challenges related to psychological distress, such as attempting suicide during the past year, as well as suicide ideation, in stark contrast to the general population [4] or other (non-bariatric) surgery candidates [5]. They suffer an increased risk of anxiety, depression, and suicidality (used hereafter to refer to suicidal ideation behavior and attempts) [6]), which may be partially attributed to a critical decline in their quality of life [7]. The psychological quality of life of bariatric surgery candidates may be damaged by body image dissatisfaction (BID)-a common psychological problem that affects many individuals in the Western world [8]. Evidence has shown that extremely obese individuals, especially females, are generally less satisfied with their bodies than the normal-weight population [3]. Furthermore, BID was found related to weight-related stigma, lower self-esteem, overt discrimination, increased symptoms of depression [9,10], anxiety [11,12], psychiatric problems, [13,14], and suicidality [15] among bariatric surgery patients. Monitoring and identifying the underlying mechanisms involved in the psychological well-being of bariatric surgery candidates is, therefore, vital, as it may improve surgery preparation and thereby promote greater satisfaction with the process.

Another important variable related to psychological distress among bariatric surgery candidates is emotional eating, namely, the tendency to eat when experiencing negative affect [16]. Emotional eating is widespread (around 40%) among bariatric surgery candidates [17] and was found associated with psychological distress symptoms [18]. According to affect regulation/escape theories, emotional eating can either reduce awareness of distress via blocking or dissociation [19] or follows such a decrease in awareness of distress [20]. It is, therefore, assumed that individuals with obesity engage in maladaptive emotional eating in order to calm themselves and to downregulate their unwanted emotions, thus creating the vicious circle of negative emotion (CODT-the circle of discontent theory) [21]. Strong empirical research (see review by Dingemans et al. [22]) has supported CODT’s assertion that the human obesity epidemic is associated with a lack of adaptive strategies to regulate negative affect and not merely with the experience of these emotions [23].

A step toward incorporating psychological well-being in the process of preparing for bariatric surgery was made in a recent Israeli study [15] that identified a positive correlation between preoperative BID and psychological distress, specifically, depressive, and anxiety symptoms and suicidality. Their findings suggested that emotional eating should be integrated in the model in order to capture the externalized and internalized weight bias stigma that affects depression and suicidality in bariatric surgery candidates. The relationship between BID and depression and suicidality was mediated by emotional eating. 

As emotional eating patterns are culture dependent [24], there is a need to test this model [15] across different cultures. Understanding the similarities and discrepancies between cultures in terms of their impact on bariatric surgery candidates is critical for adapting effective psychological interventions from one culture to another and developing personalized medicine for patients prior to surgery. Body norms and attitudes towards body weight have, in particular, been proved context dependent [25,26,27]. The United States and Israel are examples of industrialized Western nations where body fat is considered bad and there is pervasive stigmatization of overweight and obese individuals [28,29]. Nevertheless, obesity rates (the percentage of adults with a body mass index [BMI] >30) in Israel were found to be lower than those in the state of New York (16.6% [30] and 25.5% [31] in 2017, respectively). Evidence shows that the degree to which individuals feel the need to address their weight and the extent to which obesity is recognized may be affected by the regional obesity prevalence [32]. Thus, it may be that obesity may be less acceptable, and raise more concern, in Israel, than in the United States.

There are various cultural differences between the United States and Israel with regard to food and its role. US culture is individualistic [33] and characterized by food worrying, according to which food is, at times, perceived as dangerous; so that eating may be more harmful than not eating [34]. Israeli culture, on other hand, is both an industrialized Western culture and a traditional culture [35]. In traditional Jewish culture, that represents the majority of the Israeli inhabitants, there is relatively less emphasis on physical attractiveness and a strong association between food and social familial affection [36], perceived as an important source of support [37]. 

The present study aimed to broaden the examination of the associations between BID and psychological distress variables that were found among bariatric surgery candidates in Israel [15] and to compare the results with a different culture, namely, upstate New York. Specifically, we sought to examine whether cultural differences have an impact on Geller et al.’s [15] mediation model, according to which the relationship between body image and psychological distress is mediated through emotional eating.

## 2. Methods

### 2.1. Procedure and Ethics

The study was carried out in the United States and Israel between the years 2015–2017. It was part of a longitudinal study that that aimed at collecting psychosocial data from preoperative and postoperative bariatric surgery patients. The study received approval of both the IRB of the University-Based Bariatric Centers in in the United States [Mary Imogene Bassett Hospital IRB NY (Project #1074) Original approval date 07.01.2014], and in Israel [The Tel-Aviv Sourasky Medical Center (0511-13 TLV) Original approval date 27.5.14]. All respondents gave their informed consent for inclusion before they participated in the study. In the United States, patients were identified as eligible for the study once they had been approved for surgery by the Bariatric Surgery team. An appointment, at a time convenient for the patient, was arranged by the study nurse who interviewed the patients for study inclusion, obtained informed consent, and verified completion of the questionnaire prior to surgery. In Israel, each consented participant completed a self-report questionnaire, one week prior to surgery, at their pre surgery assessment. Assistance in completing the questionnaires, as well as a referral sheet with mental health services information were provided if needed. 

### 2.2. Participants 

The Israeli sample was comprised of 114 consecutive participants seeking bariatric surgery who were recruited a week prior to scheduled surgery. Sixty-six percent (66%) of the participants were females, mean age, 41.4 years (SD = 11.9, range: 18–67), mean BMI 41.3 kg/m^2^ (SD = 6.1, range: 27–71). 

The US sample was comprised of 81 consecutive participants seeking bariatric surgery who were recruited prior to scheduled surgery. Eighty percent (80%) of the participants were females, mean age, 45.8 years (SD = 11.2, range: 22–69), mean BMI 45.5 kg/m^2^ (SD = 7.5, range: 35–79). 

### 2.3. Measures

*Body Shape Questionnaire-8C* (BSQ-8C) [38]. The BSQ-8C is an 8-item questionnaire designed to evaluate participants’ dissatisfaction with their body shape. Each item, e.g. ’Have you been afraid that you might become fat (or fatter)?’ is rated on a 6-point scale, ranging from 1 *(never*) to 6 (*always*), based on the individual’s emotional state during the last 4 weeks. The total score is the sum of the 8 items, ranging from 8 to 48, and higher scores indicate higher levels of distress about body shape. Internal consistency of the measure in the current study was satisfactory (Cronbach’s *alpha* Israel = 0.82; United States = 0.77).

*Emotional Eating Scale (EES)* [16] The EES is a 25-item scale designed to assess the tendency of individuals to eat in response to negative emotional stimuli, e.g., ’furious’, ’lonely’, with three subscales: Anger/frustration, anxiety, and depression, and a total score. Each item is rated on a 5-point scale, ranging from 1 (*no desire to eat*) to 5 (*an overwhelming urge to eat*). The total score is the mean of the 25 items, ranging from 1 to 5, hence higher scores indicate a greater urge to eat in response to negative mood states. Internal consistency of the EES total in the current study was satisfactory, (Cronbach’s *alpha* IL = 0.95; United States = 0.95).

*The Suicidal Behaviors Questionnaire-Revised (SBQ-R)* [39]. A brief self-report measure of suicidal thoughts and past suicidal attempts. The 4-items assess different dimensions of suicidality: Previous suicide attempts, frequency of suicidal ideation, previous suicidal communication, and subjective likelihood of a future suicide attempt. The total score is the sum of questions ranging from 3 to 18, and higher scores indicate increased risk of suicide The internal consistency of the SBQ-R in the present study was found to be satisfactory (Cronbach’s *alpha* IL = 0.76; United States = 0.71).

The original English version of these three questionnaires were translated to Hebrew in previous studies (for details see [15]).

*Depressive severity* was assessed using the 9-item Patient Health Questionnaire (PHQ-9) [40]. The PHQ-9 is a 9-item self-report screening tool and severity measure of depression. The component scores range from 0 (*not at all*) to 3 (*nearly every day*). The column scores are added together to obtain a global score, which ranges from 0 to 27. Higher scores indicate higher levels of depression. Internal consistency of the PHQ-9 in the current study was satisfactory (Cronbach’s *alpha* IL = 0.85, United States = 0.86).

*Generalized Anxiety Disorder - Item Scale* (GAD-7) [41]. The GAD-7 is a 7-item self-report screening tool and severity measure for generalized anxiety (panic disorder, social anxiety disorder, and post-traumatic stress disorder), on a 4-point scale, from 0 (*not at all*) to 3 (*nearly every day*). The scores are added to produce a total anxiety score ranging from 0 to 21, and higher scores indicate higher/increased levels of anxiety. The internal consistency of the GAD-7 in the current study was found to be satisfactory (Cronbach’s *alpha* IL = 0.79; United States = 0.84). We used the validated Hebrew versions of both PHQ-9 [40] and GAD-7 [41].

### 2.4. Demographic Information 

Participants were asked to report basic demographic information, including gender, age, height, weight, and education. Body mass index was calculated as weight in kilograms divided by the square of height in meters (kg/m^2^).

### 2.5. Statistical Analysis

Descriptive data is presented as means and standard deviations or counts and percentages, as appropriate. One-way ANOVA was used to test for differences between the cultures in continuous measures, and the Chi-square test was used to test for differences in categorical variables. A two-way ANOVA was used to test if the differences between countries are gender dependent. Pearson correlation coefficients were calculated to evaluate the relation between the study variables in each country. Structural Equation Modeling (SEM) with bootstrapping (1000 samples) was used to test the moderated-mediation hypotheses by multi-group analysis constraining equality on structural weights. Data were analyzed using IBM SPSS Statistics v.25 and IBM SPSS Amos v.25, PA, USA. 

## 3. Results

### 3.1. Preliminary analysis 

Preliminary analysis is presented in Table 1 and in Table 2. 

As shown in Table 1, the US participants were more likely to be older females with a higher BMI, and e a higher education than the Israeli participants. 

With regards to main study variables, we found significant differences between the samples in body image and emotional eating scores, such that the US sample presented with higher BID scores, and the Israeli sample had higher emotional eating scores. No differences were found in any of the psychological distress measures. However, since suicidality correlated with gender in the US sample (Table 2), we used a two-way ANOVA to test whether the difference between the countries in suicidality is gender dependent. We found a significant gender by culture interaction (F(1, 191) = 5.0, *p* = 0.027) indicating that US men compared to US women, were significantly more inclined to suicidality, and that no difference between genders exists in the Israeli sample. 

### 3.2. Testing the Moderated Mediation Models

Geller et al. [15] found that the relationship between body image and psychological distress variables was mediated by emotional eating. We tested whether this effect could be extended to the US bariatric surgery candidates. Specifically, we used multi-group analysis to examine if the role of emotional eating as a mediator between body image and psychological distress (depression, suicidality, anxiety) is similar in both countries. 

### 3.3. Depression

**Figure 1:**
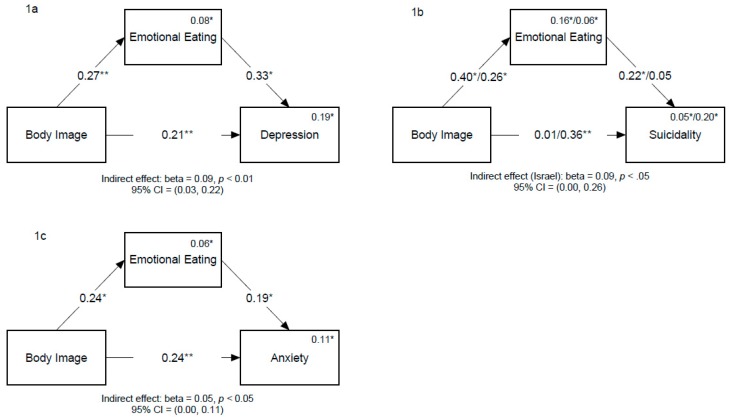
Moderated-Mediation models for depression (**1a**), suicidality (**1b**), and anxiety (**1c**) outcomes. Numbers on solid lines are standardized path coefficients for the combined Israel/US samples. *Note:* Numbers above endogenous variables names are multiple squared correlations. The analysis shown in (**1b**) was performed controlling for gender (not presented in the figure). * *p* < 0.05; ** *p* < 0.01.

We found no significant difference between cultures in the model predicting depression. Consequently, we tested the mediation model on the combined sample. We found both direct (beta = 0.21, *p* < 0.05, 95% Confidence Interval [CI] = 0.06, 0.39) and indirect (beta = 0.09, *p* < 0.01, 95% CI = 0.03, 0.22) effects (Figure 1a).

### 3.4. Suicidality

The model for predicting suicidality was tested controlling for gender as it significantly correlated with suicidality in the US sample. We found a significant difference between the cultures ((χ^2^(5) = 14.3, *p* < 0.05). Accordingly, we tested the mediation model for each culture separately. We found an indirect effect for the Israeli sample (beta = 0.09, *p* < 0.05, 95% CI = 0.002, 0.26), indicating that emotional eating mediated the correlation between BID and risk of suicide. Conversely, we found only a direct effect (beta = 0.36, *p* ≤ 0.01, 95% CI = 0.13, 0.69) for the US sample (Figure 1b).

### 3.5. Anxiety

We found no significant difference between cultures in the model predicting anxiety. Thus, we tested the mediation model on the combined sample. We found both direct (beta = 0.24, *p* = 0.008, 95% CI = 0.06, 0.42) and indirect (beta = 0.05, *p* = 0.035, 95% CI = 0.003, 0.11) effects (Figure 1c).

## 4. Discussion

Our findings further supported the hypothesis that BID is a source of psychological distress in bariatric surgery candidates [7,12,15]. In accordance with previous findings [11], we found preoperative BID to be related to psychological distress symptom measures (depression and anxiety) and suicidality in both the United States and Israel. More importantly, the relationship between BID, depression, and anxiety were mediated by emotional eating in both cultures-a finding that enhances the generalizability of the proposed model. The indirect relationship between BID and suicidality, which was mediated by emotional eating in the Israeli sample, was reflected in a direct link between BID and suicidality in the US sample. It should be noted that cultural differences were indeed manifested in elevated BID yet less emotional eating in the US sample in comparison to the Israeli sample. Thus, while culture may affect the precise nature of the mediating role of emotional eating in the association between BID and psychological distress, the proposed model may be generalized across cultures for some aspects of psychological distress. 

It is known that BID is related to overt discrimination, weight-related stigma, and increased symptoms of anxiety and depression [9,42]. These negative attitudes towards the body may lead bariatric surgery candidates to eat in order to calm or reward themselves, thus creating a vicious circle of negative emotions [21]. As candidates lack effective emotion regulation strategies, their attempts to downregulate unwanted feelings may result in unhealthy emotions such as anxiety and depression [43,44]. 

The direct link between BID and suicidality among bariatric surgery candidates in the United States may be understood in line with previous findings, according to which BID increases the propensity for individual self-harm behaviors as regulators of this negative affective state [45]. This disparity may be cautiously related to cultural dissimilarities and to the impact of culture on mood regulation activities [46]. Israeli culture is traditional, and social eating patterns are an integral part of the culture, designed to strengthen group identification [46]. Indeed, among Jewish Israelis, it is a very common practice to have large family or extended family meals every Friday and Saturday [36]. While social eating is related to a sense of belonging and group identification, it is also related to increased shame [46], known to be associated with BID, depression, and suicide ideation [47]. It may thus be hypothesized that Israeli candidates experience such feelings when eating. Therefore, emotional eating among the latter may be viewed as either a mechanism of negative emotion dysregulation [23] or a marker of dysfunctional emotion regulation strategies [48]. 

US culture, on the other hand, is dominated by individualistic eating and less social eating than in European countries such as Italy or France [49]. It may be speculated that this pattern of individualistic emotional eating enables a self-focused mood-regulatory activity [23] among US candidates in the form of frustration relief [47]. Individualistic versus social eating patterns may be one cultural difference that accounts for the dissimilarities between the two samples. These speculations emphasize the need to further investigate relations between cultural aspects, BID, emotional eating, and psychological distress among bariatric surgery patients.

As candidates do not react uniformly to bariatric surgery, there is a need for a personalized medicine which relates to specific characteristics of patients and is directed to specific diagnoses and effective treatment strategies [50]. Subsequent to our current findings, routine body image and emotional eating assessments should be considered as potential critical risk factors among patients undergoing bariatric surgery. Physicians and other health professionals in Israel and the United States are encouraged to recognize emotional eating behaviors resulting from BID, which may provide them with a sensitive instrument that enhances preparation for the procedure and optimizes satisfaction with the process [51]. From a cultural point of view, as mood regulation activities in traditional cultures are known to be more efficient than those in individualistic cultures [46], we recommend providing Israeli candidates with group mood regulation activities that are not related to eating (e.g., group physical exercise, social interaction) in order to facilitate their sense of belonging and mitigate feelings of shame [46]. The primary objective is to increase candidates’ sensitivity to their emotional experiences (shame) and typical ways of coping with these experiences (eating) in order to transform these feelings. In the United States, however, such a focus-i.e., facilitating pleasurable experiences unrelated to eating and finding healthier ways to individually relieve frustration, (e.g., leisure activities such as sport and sightseeing)-would be of merit only with regard to the relationship between BID and anxiety and depression. 

Based on the current findings, we recommend that US professionals address BID, in particular, when relating to candidates’ risk of suicide [45]. Psychological interventions, such as acceptance and commitment therapy (ACT) [52] and mindfulness therapy [53], might be effective for decreasing BID and weight-related stigma as a means of reducing psychological distress and promoting health-related behaviors in bariatric surgery candidates. 

To conclude, the present findings maintain at least some support for the assumption that Geller et al.’s model can be generalized across cultures with fine adjustment to particular cultures. Such a model may, in addition, contribute to both the preoperative assessment and preparation of bariatric surgery patients.

## 5. Strengths and Limitations

Our study is not flawless: (1) This study targeted candidates for bariatric surgery, thus, the generalizability of its findings is limited and excludes other medical conditions; (2) As the study design is cross-sectional, inferring causality is limited; (3) We could not verify that either cohort was representative of the study population, or that refusal to respond was not related to the psychological distress measures, since we do not have records of non-respondents; Additionally the large amount of missing data regarding anxiety requires that findings concerning this measure would be interpreted with caution; (4) The timeline for completing the questionnaires have differed somewhat between the Israeli and US participants, but these differences were minor enough not to affect the generalizability of the results regarding bariatric candidates; (5) Some candidates may have failed to reveal their true psychological concerns, specifically hiding suicidality, due to their motivation to pass the screening process. Nevertheless, despite these limitations and the initial screening, our sample still included a substantial number of psychologically distressed candidates, which may compensate for the aforementioned bias.

Future studies may include additional possible psychological mediators and moderators, such as shame, weight related stigma, and social support in order to increase the overall explained variations. Biochemical and nutritional measurements may also be recommended. Furthermore, the study population may also be expanded to target individuals from different cultural and sub-cultural backgrounds that will explore further the differences and similarities that exist across cultures regarding mood-regulatory eating activities. 

## Figures and Tables

**Table 1:** Sample differences in demographic and study variables. Categorical outcomes are described as count (%) and were compared using the Chi-square test. Continuous variables are described by means and standard deviations and were compared using the F test (one way ANOVA).

	Israel (*N* = 114) ^a^		United States (*N* = 81) ^b^			Effect Size
	Mean/Count (%)	Standard Deviation	Mean	Standard Deviation	F/Chi Square	Cohen’s D/V_c_
Age (years)	41.4	11.9	45.8	11.2	6.9 **	0.38
BMI (kg/m^2^)	41.3	6.1	45.5	7.5	17.5 **	0.61
Gender						
Female Male	75 (66%) 39 (34%)		65 (80%) 16 (20%)		4.9 *	0.16
Higher Education						
No Yes	65 (57%) 49 (43%)		30 (63%) 50 (27%)		7.2 **	0.19
SES						
Below Average Average Above average	28 (25%) 66 (59%) 28 (16%)		22 (28%) 51 (66%) 6 (8%)		3.0	0.20
Depression	4.1	4.3	3.2	4.1	2.1	0.21
Suicidality	3.4	1.3	3.8	1.6	3.7	0.27
Anxiety	10.6	3.2	11.2	3.2	0.9	0.19
Body Image	23.7	10.0	28.9	10.0	12.4 **	0.52
Emotional Eating	2.1	0.8	1.8	0.7	9.8 **	0.40

*Note:*^a^ Body mass index (BMI): *N* = 110, Anxiety: *N* = 63, ^b^ Age: *N* = 79, BMI: *N* = 78, Anxiety: *N* = 49. * *p* < 0.05, ** *p* < 0.01, SES = Socioeconomic status. *Note:* ^a^ Male = 2, Female = 1. ^b^ Above high school = 1, High school or less = 0. BMI = Body mass index. * *p* < 0.05, ** *p* < 0.01.

**Table 2:** Pearson correlation coefficients between the study variables for both samples. Correlations above the diagonal correspond to the Israeli sample (*N* = 114), while correlations below the diagonal correspond to the US sample (*N* = 81).

	1	2	3	4	5	6	7	8	9
1. Gender ^a^		−0.21 *	0.01	0.18	0.11	0.06	0.25	0.18	0.09
2. Age (years)	−0.12		−0.15	−0.02	−0.03	−0.05	0.00	−0.16	0.05
3. Body mass index (kg/m^2^)	−0.08	−0.03		−0.04	−0.03	0.05	0.06	−0.01	−0.09
4. Higher education ^b^	0.06	0.19	−0.20		−0.10	0.15	−0.05	0.09	0.05
5. Depression	−0.09	−0.12	0.11	0.13		0.26 **	0.80 **	0.32 **	0.35 **
6. Suicidality	−0.23 *	−0.13	0.00	0.12	0.58 **		0.32 *	0.10	0.22 *
7. Anxiety	0.08	−0.17	0.18	0.01	0.81 **	0.48 **		0.23	0.22
8. Body image	0.13	−0.28 *	0.18	−0.10	0.37 **	0.34 **	0.37 **		0.40 **
9. Emotional eating	0.21	−0.04	−0.03	0.24 *	0.43 **	0.08	0.41 **	0.26 *	

*Note:*^a^ Male = 2, Female = 1. ^b^ Above high school = 1, High school or less = 0. BMI = Body mass index. * *p* < 0.05, ** *p* < 0.01.

## References

[1] Bray G.A. (2004). Medical Consequences of Obesity. J. Clin. Endocrinol. Metab..

[2] Finucane M.M., Stevens G.A., Cowan M.J., Danaei G., Lin J.K., Paciorek C.J., Singh G.M., Gutierrez H.R., Lu Y., Bahalim A.N. (2011). National, Regional, and Global Trends in Body-Mass Index Since 1980, Systematic Analysis of Health Examination Surveys and Epidemiological Studies with 960 Country-Years and 9.1 Million Participants. Lancet.

[3] Sarwer D.B., Dilks R.J., Spitzer J.C., Cash T., Smolak L. (2011). Weight loss and changes in body image. Body Image: A Handbook of Science, Practice and Prevention.

[4] Dong C., Li W.D., Li D., Price R.A. (2006). Extreme Obesity is Associated with Attempted Suicides: Results from a Family Study. Int. J. Obes..

[5] Mather A.A., Cox B.J., Enns M.W., Sareen J. (2009). Associations of Obesity with Psychiatric Disorders and Suicidal Behaviors in a Nationally Representative Sample. J. Psychosom. Res..

[6] Chen E.Y., Fettich K.C., McCloskey M.S. (2012). Correlates of Suicidal Ideation and/or Behavior in Bariatric-Surgery-Seeking Individuals with Severe Obesity. Crisis.

[7] Sarwer D.B., Bishop-Gilyard C.T., Carvajal R., Still C.D., Sarwer D.B., Blankenship J. (2014). Quality of life. The ASMBS Textbook of Bariatric Surgery, Vol. 2: Integrated Health.

[8] Padgett J., Biro F.M. (2003). Different Shapes in Different Cultures: Body Dissatisfaction, Overweight, and Obesity in African-American and Caucasian Females. J. Pediatr. Adolesc. Gynecol..

[9] Sarwer D., Allison K.C., Bailer B.A., Faulconbridge L.F., Still C.D., Sarwer D.B., Blankenship J. (2014). Psychosocial characteristics of bariatric surgery candidates. The ASMBS Textbook of Bariatric Surgery, Vol. 2: Integrated Health.

[10] Petry N.M., Barry D., Pietrzak R.H., Wagner J.A. (2008). Overweight and Obesity are Associated with Psychiatric Disorders: Results from the National Epidemiologic Survey on Alcohol and Related Conditions. Psychosom. Med..

[11] Rosenberger P.H., Henderson K.E., Grilo C.M. (2006). Correlates of Body Image Dissatisfaction in Extremely Obese Female Bariatric Surgery Candidates. Obes. Surg..

[12] Pona A.A., Marek R.J., Heinberg L.J., Lavery M., Ashton K., Merrell Rish J. (2017). Psychological Correlates of Body Image Dissatisfaction Before and After Bariatric Surgery. Bariatr. Surg. Pract. Patient Care.

[13] Sarwer D.B., Fabricatore A.N. (2008). Psychiatric Considerations of the Massive Weight Loss Patient. Clin. Plast. Surg..

[14] Neumark-Sztainer D., Haines J., Thompson J.K. (2004). Psychosocial and behavioral consequences of obesity. Handbook of Eating Disorders and Obesity.

[15] Geller S., Levy S., Goldzweig G., Hamdan S., Manor A., Dahan S., Rothschild E., Stukalin Y., Abu-Abeid S. (2019). Psychological Distress Among Bariatric Surgery Candidates: The Roles of Body Image and Emotional Eating. Clin. Obes..

[16] Arnow B., Kenardy J., Agras W.S. (1995). The Emotional Eating Scale: The Development of a Measure to Assess Coping with Negative Affect by Eating. Int. J. Eat. Disord..

[17] Konttinen H., Silventoinen K., Sarlio-Lähteenkorva S., Männist Ö.S., Haukkala A. (2010). Emotional Eating and Physical Activity Self-Efficacy as Pathways in the Association Between Depressive Symptoms and Adiposity Indicators. Am. J. Clin. Nutr..

[18] Van Strien T., Schippers G.M., Cox W.M. (1995). On the Relationship Between Emotional and External Eating Behavior. Addict. Behav..

[19] Hallings-Pott C., Waller G., Watson D., Scragg P. (2006). State Dissociation in Bulimic Eating Disorders: An Experimental Study. Int. J. Eat. Disord..

[20] Heatherton T.F., Baumeister R.F. (1991). Binge Eating as Escape from Self-Awareness. Psychol. Bull..

[21] Marks D.F. (2015). Homeostatic Theory of Obesity. Health Psychol. Open.

[22] Dingemans A., Danner U., Parks M. (2017). Emotion Regulation in Binge Eating Disorder: A Review. Nutrients.

[23] Evers C., Marijn Stok F., de Ridder D.T. (2010). Feeding Your Feelings: Emotion Regulation Strategies and Emotional Eating. Pers. Soc. Psychol. Bull..

[24] Luomala H., Sirieix L., Tahir R. (2009). Exploring Emotional-Eating Patterns in Different Cultures: Toward a Conceptual Framework Model. J. Int. Consum. Mark..

[25] Council S.K., Placek C. (2014). Cultural Change and Explicit Anti-Fat Attitudes in a Developing Nation: A Case Study in Rural Dominica. Soc. Med..

[26] Puraikalan Y. (2018). Obesity: Perceptions of Body Image and Obesity Among Cross Culture: A Review. Obes. Res. Open J..

[27] Davidson M., Knafl K.A. (2006). Dimensional Analysis of the Concept of Obesity. J. Adv. Nurs..

[28] Brewis A.A., Wutich A., Falletta-Cowden A., Rodriguez-Soto I. (2011). Body Norms and Fat Stigma in Global Perspective. Curr. Anthrop..

[29] Feinson M.C., Meir A. (2012). Disordered Eating and Religious Observance: A Focus on Ultra-Orthodox Jews in an Adult Community Study. Int. J. Eat. Disord..

[30] OECD Obesity Update 2017. https://www.oecd.org/els/health-systems/Obesity-Update-2017.pdf.2017.

[31] CDC National Center for Health Statistics (NCHS) Data Adult Obesity Facts 2017. https://www.cdc.gov/obesity/data/adult.html/prevalence-maps.html.

[32] Wardle J., Haase A.M., Steptoe A. (2006). Body Image and Weight Control in Young Adults: International Comparisons in University Students from 22 Countries. Int. J. Obes..

[33] Hofstede G., Hofstede G.J., Minkov M. (2010). Cultures and Organizations: Software of the Mind.

[34] Levenstein H. (2012). Fear of Food: A History of Why We Worry About What We Eat.

[35] Lavee Y., Katz R. (2003). The Family in Israel: Between Tradition and Modernity. Marriage Fam. Rev..

[36] Latzer Y., Orna T., Gefen S. (2007). Level of Religiosity and Disordered Eating Psychopathology Among Modern-Orthodox Jewish Adolescent girls in Israel. Int. J. Adolesc. Med. Health.

[37] Maor M. (2013). “Do I Still Belong Here?” The Body’s Boundary Work in the Israeli Fat Acceptance Movement. Soc. Mov. Stud..

[38] Evans C., Dolan B. (1993). Body Shape Questionnaire: Derivation of Shortened “Alternate Forms”. Int. J. Eat. Disord..

[39] Osman A., Bagge C.L., Gutierrez P.M., Konick L.C., Kopper B.A., Barrios F.X. (2001). The Suicidal Behaviors Questionnaire—Revised (SBQ—R). Validation with Clinical and Nonclinical Samples. Assessment.

[40] Kroenke K., Spitzer R.L., Williams J.B. (2001). The PHQ-9: Validity of a Brief Erity Measure. J. Gen. Int. Med..

[41] Spitzer R.L., Kroenke K., Williams J.B., Lowe B. (2006). A Brief Measure for Assessing Generalized Anxiety Disorder: The GAD-7. Arch. Int. Med..

[42] Sarwer D.B., Thompson J.K., Cash T.F. (2005). Body Image and Obesity in Adulthood. Psychiatr. Clin. N. Am..

[43] O’Brien K.S., Latner J.D., Puhl R.M., Vartanian L.R., Giles C., Griva K., Carter A. (2016). The Relationship Between Weight Stigma and Eating Behavior is Explained by Weight Bias Internalization and Psychological Distress. Appetite.

[44] Baldofski S., Rudolph A., Tigges W., Herbig B., Jurowich C., Kaiser S., Dietrich A., Hilbert A. (2016). Weight Bias Internalization, Emotion Dysregulation, and Non-Normative Eating Behaviors in Prebariatric Patients. Int. J. Eat. Disord..

[45] Muehlenkamp J.J. (2012). Body Regard in Nonsuicidal Self-Injury: Theoretical Explanations and Treatment Directions. J. Cogn. Psychother..

[46] Luomala H.T., Kumar R., Worm V., Singh J.D. (2004). Cross-Cultural Differences in Mood-Regulation: An Empirical Comparison of Individualistic and Collectivistic Cultures. J. Int. Consum. Mark..

[47] Menzel J.E., Schaefer L.M., Burke N.L., Mayhew L.L., Brannick M.T., Thompson J.K. (2010). Appearance-Related Teasing, Body Dissatisfaction, and Disordered Eating: A Meta-Analysis. Body Image.

[48] Leehr E.J., Krohmer K., Schag K., Dresler T., Zipfel S., Giel K.E. (2015). Emotion Regulation Model in Binge Eating Disorder and Obesity: A Systematic Review. Neurosci. Biobehav. Rev..

[49] Fischler C. (2011). Commensality, Society and Culture. Soc. Sci. Inf..

[50] Hommel K.A., Herzer M., Ingerski L.M., Hente E., Denson L.A. (2011). Individually Tailored Treatment of Medication Nonadherence. J. Pediatr. Gastroenterol. Nutr..

[51] Chesler B.E. (2012). Emotional Eating: A Virtually Untreated Risk Factor for Outcome Following Bariatric Surgery. Sci. World J..

[52] Levin M.E., Potts S., Haeger J., Lillis J. (2017). Delivering Acceptance and Commitment Therapy for Weight Self-Stigma Through Guided Self-Help: Results from an Open Pilot Trial. Cogn. Behav. Pract..

[53] Lillis J., Hayes S.C., Bunting K., Masuda A. (2009). Teaching Acceptance and Mindfulness to Improve the Lives of the Obese: A Preliminary Test of a Theoretical Model. Ann. Behav. Med..

